# Intravoxel incoherent motion diffusion-weighted imaging in evaluating preoperative staging of esophageal squamous cell carcinoma: Evaluation of preoperative stage of primary tumour and prediction of lymph node metastases from esophageal cancer using IVIM: a prospective study

**DOI:** 10.1186/s40644-024-00765-w

**Published:** 2024-08-29

**Authors:** Tao Song, Shuang Lu, Jinrong Qu, Hongkai Zhang, Zhaoqi Wang, Zhengyan Jia, Hailiang Li, Yan Zhao, Jianjun Qin, Wen Feng, Shaoyu Wang, Xu Yan

**Affiliations:** 1 Department of Radiology, Affiliated Cancer Hospital of Zhengzhou University & Henan Cancer Hospital, Zhengzhou, 450008 China; 2 Department of Thoracic Surgery, Affiliated Cancer Hospital of Zhengzhou University & Henan Cancer Hospital, Zhengzhou, 450008 China; 3 Department of Pathology, Affiliated Cancer Hospital of Zhengzhou University & Henan Cancer Hospital, Zhengzhou, 450008 China; 4 MR Scientific Marketing, Siemens Healthineers, XI’an, 710065 China; 5 MR Scientific Marketing, Siemens Healthineers, Shanghai, 201318 China; 6 Henan Province, 127 Dongming road, Jinshui District, Zhengzhou city, 450008 China

**Keywords:** Diffusion magnetic resonance imaging, Intravoxel incoherent motion, Esophageal cancer, Tumor staging

## Abstract

**Background:**

The aim of this research is to prospectively investigate the diagnostic performance of intravoxel incoherent motion (IVIM) using the integrated slice-specific dynamic shimming (iShim) technique in staging primary esophageal squamous cell carcinoma (ESCC) and predicting presence of lymph node metastases from ESCC.

**Methods:**

Sixty-three patients with ESCC were prospectively enrolled from April 2016 to April 2019. MR and IVIM using iShim technique (b = 0, 25, 50, 75, 100, 200, 400, 600, 800 s/mm^2^) were performed on 3.0T MRI system before operation. Primary tumour apparent diffusion coefficient (ADC) and IVIM parameters, including true diffusion coefficient (D), pseudodiffusion coefficient (D^*^), pseudodiffusion fraction (f) were measured by two independent radiologists. The differences in D, D^*^, f and ADC values of different T and N stages were assessed. Intraclass correlation coefficients (ICCs) were calculated to evaluate the interobserver agreement between two readers. The diagnostic performances of D, D^*^, f and ADC values in primary tumour staging and prediction of lymph node metastasis of ESCC were determined using receiver operating characteristic (ROC) curve analysis.

**Results:**

The inter-observer consensus was excellent for IVIM parameters and ADC (D: ICC = 0.922; D^*^: ICC = 0.892; f: ICC = 0.948; ADC: ICC = 0.958). The ADC, D, D^*^ and f values of group T1 + T2 were significantly higher than those of group T3 + T4a [ADC: (2.55 ± 0.43) ×10^− 3^ mm^2^/s vs. (2.27 ± 0.40) ×10^− 3^ mm^2^/s, *t* = 2.670, *P* = 0.010; D: (1.82 ± 0.39) ×10^− 3^ mm^2^/s vs. (1.53 ± 0.33) ×10^− 3^ mm^2^/s, *t* = 3.189, *P* = 0.002; D^*^: 46.45 (30.30,55.53) ×10^− 3^ mm^2^/s vs. 32.30 (18.60,40.95) ×10^− 3^ mm^2^/s, *z*=-2.408, *P* = 0.016; f: 0.45 ± 0.12 vs. 0.37 ± 0.12, *t* = 2.538, *P* = 0.014]. The ADC, D and f values of the lymph nodes-positive (N+) group were significantly lower than those of lymph nodes-negative (N0) group [ADC: (2.10 ± 0.33) ×10^− 3^ mm^2^/s vs. (2.55 ± 0.40) ×10^− 3^ mm^2^/s, *t*=-4.564, *P* < 0.001; D: (1.44 ± 0.30) ×10^− 3^ mm^2^/s vs. (1.78 ± 0.37) ×10^− 3^ mm^2^/s, *t*=-3.726, *P* < 0.001; f: 0.32 ± 0.10 vs. 0.45 ± 0.11, *t*=-4.524, *P* < 0.001]. The combination of D, D^*^ and f yielded the highest area under the curve (AUC) (0.814) in distinguishing group T1 + T2 from group T3 + T4a. D combined with f provided the highest diagnostic performance (AUC = 0.849) in identifying group N + and group N0 of ESCC.

**Conclusions:**

IVIM may be used as an effective functional imaging technique to evaluate preoperative stage of primary tumour and predict presence of lymph node metastases from ESCC.

## Background

Esophageal cancer (EC) is the seventh most common malignancy in the world, with an overall 5-year survival rate of 10–30% [1]. At present, the treatment of EC is strongly dependent on accurate clinical staging, especially the depth of infiltration and lymph node involvement, which are also the most important prognostic factors in EC [2, 3]. Therefore, precise pretreatment staging is extremely imperative to determine the optimal treatment opinion and influence the prognosis.

Currently, the clinical staging of EC mainly depends on traditional imaging modalities such as multidetector computed tomography (MDCT), endoscopic ultrasonography (EUS) and magnetic resonance imaging (MRI). EUS is currently considered the preferred imaging work-up for T staging of EC, reported accuracy rates are widely variable, ranging between 64% and 92% [4–6]. Additionally, EUS is an invasive diagnostic work-up, which cannot be performed when the esophagus is completely obstructed by the tumor. MDCT has a limited utility for T staging because the technique cannot precisely differentiate esophageal wall layers, along with the use of ionizing radiation. Previous studies have reported that the accuracy of MDCT for T staging is lower than that of EUS [7, 8]. PET/CT is not primarily used for T staging of the primary tumor due to its poor spatial resolution [9]. In recent years, MR is increasingly used in the staging of EC [10, 11]. However, due to the limitation of spatial resolution, the display of the very thin esophageal adventitia is still unsatisfactory, which can lead to overestimation or underestimation of T staging.

Traditional imaging modalities mainly depend on size criteria to identify pathologic lymph nodes, however, this is not a reliable criterion [8]. PET/CT has high diagnostic specificity but low sensitivity, and the specificity and sensitivity for detecting regional lymph node involvement further decreases in smaller lymph nodes. Given the stated limitations of each imaging technique, none can be used for satisfactory evaluating lymph node metastases [5, 12, 13].

Although the accuracy of conventional imaging modalities in the preoperative staging of EC continues to increase, about 20% of early-stage esophageal cancers are still found after resection to be more advanced than preoperative staging [14]. Therefore, it is necessary to identify some biomarkers to assist in evaluating the clinical staging of EC.

Currently, DWI has been widely used in tumor detection, characterization and monitoring [5, 15–18]. Whereas, the DWI-derived apparent diffusion coefficient (ADC) cannot differentiate perfusion from diffusion effects, and its value in the preoperative staging of EC is still controversial [5, 17]. Unlike ADC, the advantage of intravoxel incoherent motion (IVIM) is that it can separate and quantify microcirculatory perfusion and molecular diffusion through the acquisition parameters: true diffusion coefficient (D), pseudodiffusion coefficient (D^*^) and pseudodiffusion fraction (f) [19]. IVIM is gradually being applied to clinical research and has been proven to be more valuable and promising than ADC in tumor monitoring and assessing treatment response [20–24]. To date, few researches have assessed the application of IVIM sequence in the preoperative staging of esophageal squamous cell carcinoma (ESCC) [25, 26].

According to previous literature [27–29], the integrated slice-specific dynamic shimming (iShim) technique can reduce the geometric deformation, displacement artifacts and increase the signal-to-noise ratio (SNR), which can improve the accuracy of IVIM parameters fitting and provide excellent inter-observer reproducibility on IVIM parameters. To our knowledge, IVIM using iShim technique has not been used for the preoperative staging of ESCC.

Therefore, our research aimed to prospectively investigate the potential value of iShim IVIM-derived parameters in staging primary ESCC and predicting presence of lymph node metastases from ESCC.

## Materials and methods

### Study population

This prospective research was approved by the Ethics Committee of our Hospital and signed written informed consent was obtained from all participating patients. Between April 2016 and April 2019, a total of 133 consecutive patients with EC confirmed by endoscopic pathology were prospectively enrolled.

The inclusion criteria were as follows: (1) histological diagnosis of ESCC; (2) potentially resectable stage of T and N assessed by conventional imaging modalities; (3) All patients had undergone MRI and IVIM examination before surgical resection; (4) without previous history of any malignancy or tumor-related therapy; (5) radical esophagectomy, with histopathological evaluation.

The exclusion criteria were: (1) MRI contraindications (*n* = 1); (2) patients treated with neoadjuvant chemo-/radiotherapy (*n* = 61); (3) inferior quality of IVIM images (e.g., numerous motion artifacts) was insufficient for further analysis (*n* = 5); (4) the tumor was too small to draw the region of interest (ROI) (*n* = 3).

### MRI protocol and imaging analysis

All patients underwent MR and IVIM examination within one week before radical esophagectomy. MRI and IVIM examinations were performed on a 3.0 T MR system (MAGNETOM Skyra, Siemens Healthcare) with an anterior 18-element body coil and in-built posterior 32-element spine coil array. The routine MRI and IVIM protocol covered the chest. IVIM scan ranged from the thoracic entrance level to the costophrenic angle level. All patients were instructed on free-breathing and breath-holding techniques before image acquisition to reduce motion artifacts. Raceanisodamine hydrochloride injection (Dahongying Pharmaceutical Co.) with a dose of 10 mg was injected intramuscularly 15–20 min before MRI and IVIM examination to inhibit the peristalsis of the esophagus.

The MRI protocol included axial T2-weighted images using turbo spin-echo (TSE) BLADE (a product of Siemens Healthcare) sequence, axial T1-weighted images using volumetric interpolated breath hold examination (VIBE) sequence, and IVIM sequence using iShim technique. The detailed MRI parameters for each sequence are listed in Table 1. The acquisition times of axial T1WI and T2WI are 18 s and 4–6 min, respectively. IVIM was acquired by using nine b-values of 0, 25, 50, 75, 100, 200, 400, 600, and 800 s/mm^2^. The total acquisition time of IVIM sequence was approximately 3:30 min.

**Table 1:** MRI parameters

Parameters	Axial T1-weighted VIBE	Axial T2-weighted TSE BLADE	IVIM-iShim
TR (ms)	2.72	5000	3200
TE (ms)	0.95	97	56
Slice thickness (mm)	3.0	3.0	5.0
NEX	1	1	1(b = 0–200 s/mm^2^); 2(b = 400–600 s/mm^2^); 3(b = 800 s/mm^2^)
FOV (mm^2^)	360 × 360	240 × 240	340 × 340
Matrix	224 × 384	256 × 256	128 × 128

TR = repetition time; TE = echo time; NEX = number of excitations; FOV = field of view; VIBE = volumetric interpolated breath hold examination; TSE = turbo spin-echo; BLADE = a product of Siemens Healthcare; iShim = integrated specific slice dynamic Shim

All original IVIM data was loaded to the workstation and processed by MADC software package. All IVIM images were analyzed separately by two radiologists (S T with sixteen years of experience in digestive radiology and W Z Q with seven years of experience in MRI), who were blinded to the pathological consequence. With reference to axial T2WI, ROI was manually drawn on the IVIM imaging with a b-value of 400 s/mm^2^ by the previous two radiologists independently, and the axial slice with the maximum primary ESCC dimension was contoured as the ROI, excluding the esophageal cavity and areas of cystic or necrotic degeneration, with an area of ​​at least 50 mm^2^. The pseudocolor maps of D, D^*^, f and ADC were generated automatically by MADC software. The ROI was transferred to the corresponding pseudocolor maps. The values of IVIM-derived parameters (D, D^*^ and f) were automatically measured by the bi-exponential model. The measurement of ADC value was performed on the image with a b value of 0 s/mm^2^ and 800 s/mm^2^ using the monoexponential model.

### Pathologic examination

Based on the 8th edition of the American Joint Committee on Cancer (AJCC) TNM cancer categories for esophageal cancer [30], T and N staging of surgically resected specimens were assessed by a pathologist with 15 years of experience, who was blinded to the IVIM data.

### Statistical analysis

All statistical analyses were performed by using SPSS version 22.0 (SPSS Inc., Chicago, IL). The inter-observer consensus for D, D^*^, f and ADC was assessed by calculating the intraclass correlation coefficient (ICC) (0.81–1.00, excellent correlation; 0.61–0.80, good correlation; 0.41–0.60, moderate correlation; 0.21–0.40, fair correlation; 0.00–0.20, poor correlation) [31]. Kolmogorov-Smirnov test assessed whether the data follow the normal distribution. Continuous variables were expressed as mean ± standard deviation, and measurement data that do not follow the normal distribution were represented as the median (upper and lower quartile).

Independent sample *t*-test and Wilcoxon rank-sum test were used to analyze the differences of D, D^*^, f and ADC values between different T stages and lymph node status. Receiver operating characteristic (ROC) curve analyses were performed to evaluate the diagnostic performance of the parameters (D, D^*^, f and ADC) in distinguishing different depths of local invasion and lymph node status. The area under the curve (AUC), sensitivity and specificity were calculated. *P* < 0.05 was considered statistically significant.

## Results

### Study population

A total of 133 patients were consecutively enrolled in this research. According to the exclusion criteria, sixty-three patients were finally enrolled. Patients had a mean age of 60.3 ± 9.1 years (age range: 42–71years), and forty-one of them were male.

Eight lesions were located in the upper thoracic esophagus, 42 were located in the middle thoracic part, and 13 were located in the lower thoracic part. The pathological type was all ESCC.

**Fig. 1:**
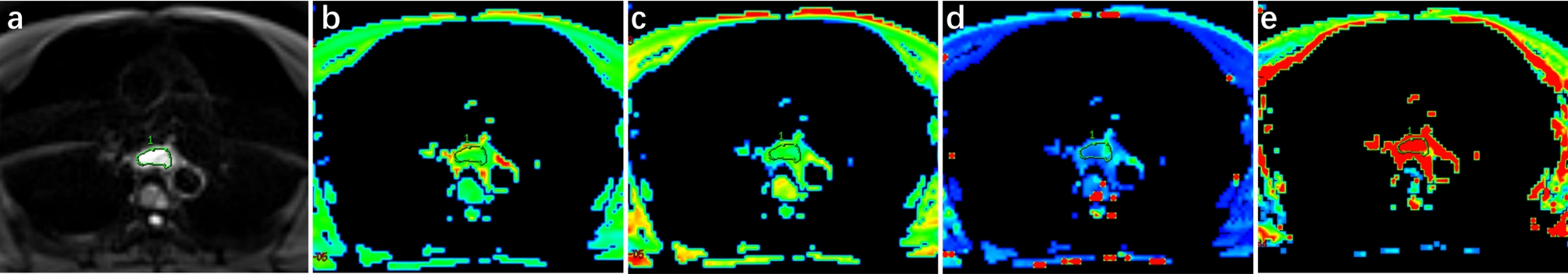
A 56-year-old man with a mid-esophageal squamous cell carcinoma with pathological staging T3N1. **a** Axial diffusion-weighted image (b = 400 s/mm^2^) shows a hyperintense focal esophageal lesion. The region of interest (ROI) (green contours) was manually delineated on axial sections of the images. **b-e** The corresponding ADC, D, D^*^and f maps show the ADC value of 1.95 × 10^− 3^ mm^2^/s, a D value of 1.09 × 10^− 3^ mm^2^/s, a D^*^ value of 19.5 × 10^− 3^ mm^2^/s and f value of 0.37 of the lesion

**Fig. 2:**
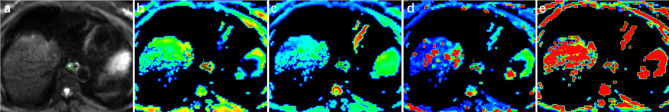
A 61-year-old woman with a lower thoracic esophageal squamous cell carcinoma with pathological staging T2N0. **a** Axial diffusion-weighted image (b = 400 s/mm^2^) shows a hyperintense focal esophageal lesion. The region of interest (ROI) (green contours) was manually delineated on axial sections of the images. **b-e** The corresponding ADC, D, D^*^and f maps show the ADC value of 2.30 × 10^− 3^ mm^2^/s, a D value of 1.88 × 10^− 3^ mm^2^/s, a D^*^ value of 93.6 × 10^− 3^ mm^2^/s and f value of 0.47 of the lesion

Based on the 8th edition of the AJCC TNM cancer categories for esophageal cancer, only one case was stage T1, 25 cases were stage T2, 36 cases were stage T3, and 1 case was stage T4a. Lymph nodes-negative (N0) group included 40 cases. The regional lymph nodes-positive (N+) group included N1 (16 cases) and N2 (7 cases), a total of 23 cases. Typical IVIM images for different pathologic T and N staging of ESCC are presented in Figs. 1 and 2.

### Inter-observer consensus of IVIM parameters and ADC

The inter-observer consensus was excellent for IVIM parameters (D, D^*^ and f) and ADC of ESCC (D: ICC = 0.922; D^*^: ICC = 0.892; f: ICC = 0.948; ADC: ICC = 0.958).

### Comparison of ADC and IVIM parameters between group T1 + T2 and group T3 + T4a

Since there was only one patient in T1 and T4a respectively, according to whether the adventitia was invaded, patients were divided into two groups, namely non-invasion of adventitia group (T1 + T2) and invasion of adventitia group (T3 + T4a). The results confirmed that the D, D^*^, f and ADC values of group T1 + T2 were significantly higher than those of group T3 + T4a of ESCC (detailed values in Table 2).

**Table 2:** Comparison of ADC and IVIM parameters between group T1 + T2 and group T3 + T4a

T stage	Case (*n*)	ADC (×10^− 3^ mm^2^/s)	D (×10^− 3^ mm^2^/s)	D^*^ (×10^− 3^ mm^2^/s)		f
T1 + T2	26	2.55 ± 0.43	1.82 ± 0.39	46.45 (30.30,55.53)	0.45 ± 0.12	
T3 + T4a	37	2.27 ± 0.40	1.53 ± 0.33	32.30 (18.60,40.95)	0.37 ± 0.12	
*t* value		2.670	3.189	-2.408^*^	2.538	
*P* value		0.010	0.002	0.016	0.014	

^*^: *Z* value; ADC = apparent diffusion coefficient; IVIM = intravoxel incoherent motion

### ROC analyses of ADC and IVIM parameters to T stage of ESCC

**Fig. 3:**
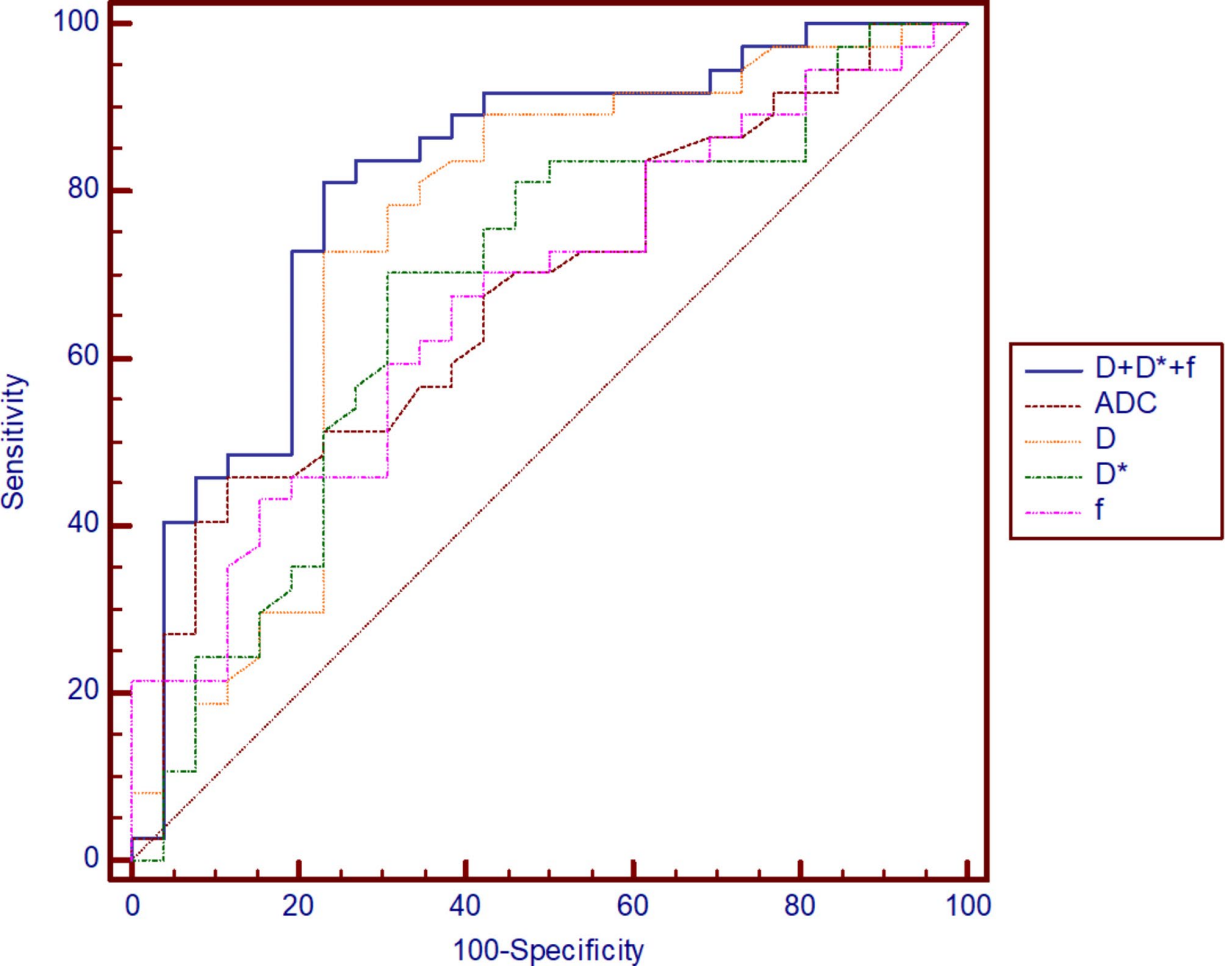
The ROC show the utility of IVIM parameters and ADC to discriminate group T1 + T2 from group T3 + T4a of esophageal squamous cell carcinoma

As shown in Fig. 3, the above parameters with statistical differences were furtherly performed by ROC curve analysis to evaluate the performance in distinguishing group T1 + T2 from group T3 + T4a. The best cut-off values, sensitivity, specificity and the AUC were calculated (detailed values in Table 3). Especially, D combined with D^*^ and f could identify whether the adventitia was invaded, yielding an AUC of 0.814 for differentiating group T1 + T2 from group T3 + T4a.

**Table 3:** The diagnostic performance of IVIM parameters and ADC in discriminating group T1 + T2 from group T3 + T4a

Parameters	Cut-off value	Sensitivity	Specificity	Youden index	AUC
ADC (×10^− 3^ mm^2^/s)	2.13	45.9%	88.5%	0.344	0.678
D (×10^− 3^ mm^2^/s)	1.70	73.0%	76.9%	0.499	0.739
D^*^ (×10^− 3^ mm^2^/s)	34.90	70.3%	69.2%	0.395	0.679
f	0.41	67.6%	61.5%	0.291	0.671
D + D^*^+f	0.53	81.1%	76.9%	0.580	0.814

IVIM = intravoxel incoherent motion; ADC = apparent diffusion coefficient; AUC = area under the curve

### Comparison of ADC and IVIM parameters between group N + and group N0

The results demonstrated that D, f and ADC values of group N + were significantly lower than those of group N0 (all *P <* 0.01). In addition, no differences in D^*^ could be found between two groups (*P* > 0.05). The detailed IVIM parameters and ADC values ​​were shown in Table 4.

**Table 4:** Comparison of ADC and IVIM parameters between group N + and group N0

*N* stage	Case (*n*)	ADC (×10^− 3^ mm^2^/s)	D (×10^− 3^ mm^2^/s)	D^*^ (×10^− 3^ mm^2^/s)	f
N+	23	2.10 ± 0.33	1.44 ± 0.30	32.10 (15.40,49.90)	0.32 ± 0.10
N0	40	2.55 ± 0.40	1.78 ± 0.37	35.35 (26.83,50.20)	0.45 ± 0.11
*t* value		-4.564	-3.726	-1.371^*^	-4.524
*P* value		< 0.001	< 0.001	0.170	< 0.001

^*^: *Z* value; ADC = apparent diffusion coefficient; IVIM = intravoxel incoherent motion

### ROC analyses of ADC and IVIM parameters to lymph node metastases of ESCC

**Fig. 4:**
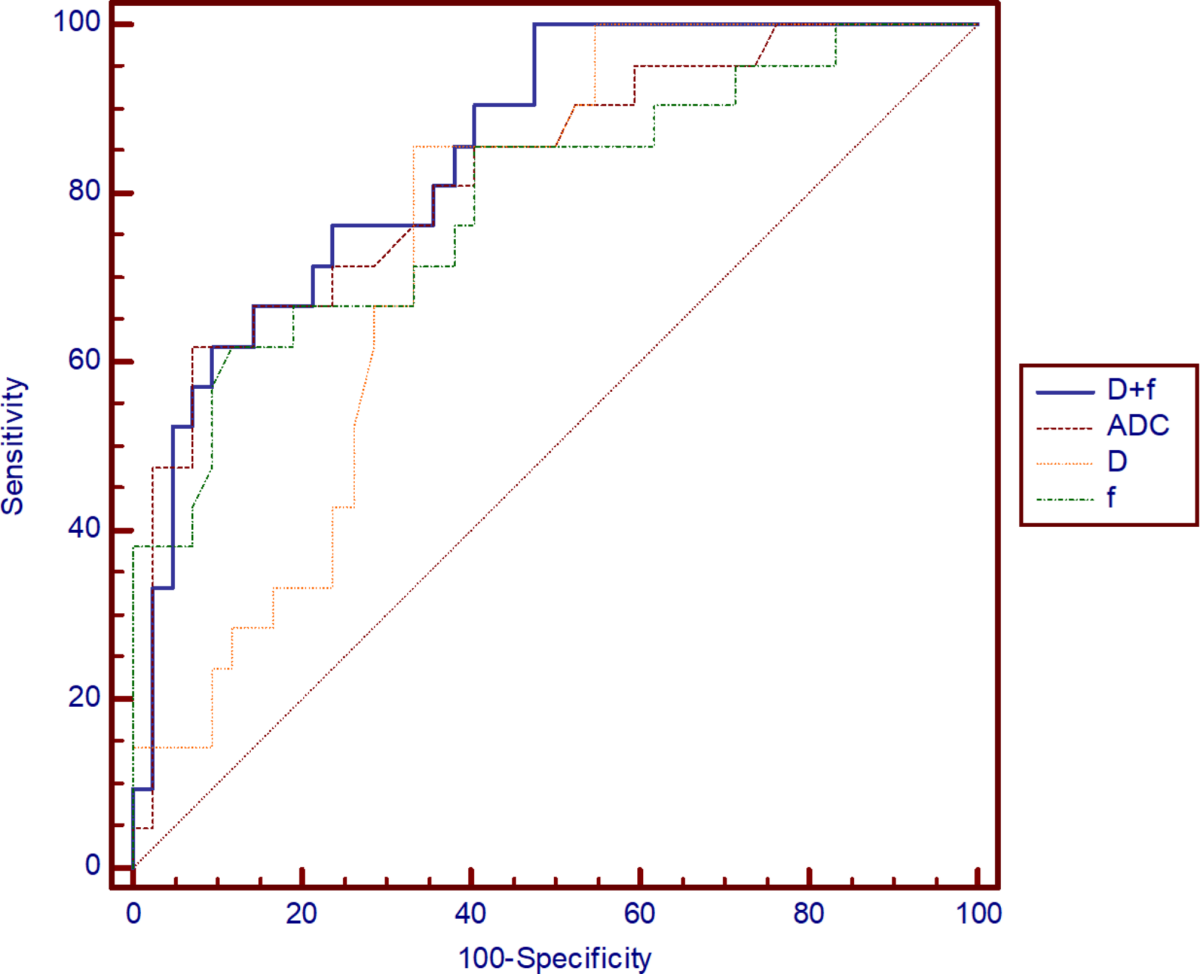
The ROC show the utility of IVIM parameters and ADC to discriminate group N + from group N0 of esophageal squamous cell carcinoma

As shown in Fig. 4, the above parameters with statistical differences were furtherly performed by ROC curve analysis to assess the diagnostic performance in distinguishing N0 from N + in ESCC. The best cut-off values, sensitivity, specificity and the AUC were calculated (detailed values in Table 5). Especially, D combined with f could identify ESCC with and without lymph node involvement, with a sensitivity of 100.0% and a specificity of 52.4% (AUC = 0.849).

**Table 5:** The diagnostic performance of IVIM parameters and ADC in discriminating group N + from group N0

Parameters	Cut-off value	Sensitivity	Specificity	Youden index	AUC
ADC (×10^− 3^ mm^2^/s)	2.04	61.9%	92.9%	0.547	0.822
D (×10^− 3^ mm^2^/s)	1.68	85.7%	66.7%	0.523	0.750
f	0.32	61.9%	88.1%	0.500	0.794
D + f	0.15	100.0%	52.4%	0.523	0.849

IVIM = intravoxel incoherent motion; ADC = apparent diffusion coefficient; AUC = area under the curve

## Discussion

Since signal properties on functional imaging modalities reflect the microstructure and the physiologic status of tissue independent from the morphological feature of the tumor, they can overcome the limitations of conventional imaging examination and play an important role in the clinic. Our study has demonstrated that the feasibility and application value of IVIM parameters in preoperative evaluation of T staging and lymph node metastases of ESCC, and the diagnostic efficiency of combined IVIM parameters (D, D^*^ and f) is higher than that of individual parameter. The study results suggested that ESCC with lower values of IVIM-derived parameters (D, D^*^ and f) and ADC had a trend to be at advanced depth of tumor invasion (T staging) and lymph node metastases. The possible reason is that with the progress of ESCC, the decrease in the number of keratinization and the increase of the proportion of basaloid cells lead to the restriction of diffusion [32], so the D and ADC values decrease. As the number of tumor cells increases, the extracellular space decreases, causing an increase of intratumoral interstitial hypertension, which leads to a decrease in microcirculatory perfusion. In clinical practice, we suggest that the combination of conventional MR and IVIM in preoperative examination of ESCC, combined with morphology and function, can provide more effective, additional information for accurate preoperative staging in ESCC.

In this study, IVIM sequence using iShim technique was performed to improve the accuracy and stability of IVIM parameters. The results of our research demonstrated that the inter-observer consensus was excellent for IVIM parameters and ADC values. Our previous studies had also confirmed that the application of IVIM using the iShim technique in ESCC was reliable and valuable [22].

To date, the role of IVIM in ESCC is unclear and not yet thoroughly investigated [33]. A previous study showed no difference in IVIM parameters between EC and normal esophageal tissue [34]. Recent studies have indicated that the ADC value was contributing to assess the T stage of EC, but its performance in the N stage is still controversial [5, 17, 25]. In our study, the D value exhibited better performance than conventional DWI. With the D value of 1.70 × 10^− 3^ mm^2^/s as the threshold, the diagnostic efficacy is superior to the ADC, D^*^ and f values for assessing T staging of ESCC. Especially the combination of D, D^*^ and f yielded the highest diagnostic performance in distinguishing stage T1 + T2 from stage T3 + T4a. Compared to conventional DWI, IVIM uses the biexponential model, which can differentiate and quantify true water molecular diffusion and microvascular perfusion. Hence, in comparison with ADC, the D value may more precisely characterize the actual status of perfusion and diffusion of tumors. Similar to our research, a study using IVIM to assess the staging of bladder cancer showed that the D value revealed better diagnostic performance than the ADC value for distinguishing different T stages [35]. However, a previous study demonstrated that the performance of f value is better than any other IVIM-derived parameters for identifying the T stage of ESCC [25]. In our research, we confirmed that the combination of D, f and D^*^ yielded the highest AUC (0.814) and sensitivity (81.1%), which might aid in more accurately differentiating T-stage of ESCC. In short, ESCC with lower values of IVIM parameters (D, f and D^*^) were associated with advanced T staging. A previous study reported that a negative correlation between IVIM parameters and staging of gastro-esophageal cancer [33], and Mizumachi, R et al. confirmed that lower ADC and perfusion-related parameter values of the tumor were significantly associated with the higher clinical T stage of ESCC, which is consistent with our results [26].

Due to the limitation of spatial resolution and size criteria, lymph node involvement in ESCC cannot be precisely determined on the traditional imaging examination. Our study confirmed that D, f and ADC values could be effective in preoperative assessment of the lymph node status of ESCC. The combination of D and f values yielded the highest AUC (0.849) in identifying ESCC with and without lymph node involvement, and the sensitivity could reach 100%. Based on the results of previous studies [32, 36], as the progress of tumor, the restriction of molecular diffusion of the tumor tissue increases and the microvascular perfusion decreases, which may explain that the D value, f value and ADC value of ESCC with lymph node metastasis are lower than those without lymph node metastasis. Similar findings have been reported in other tumors. Several studies have shown that IVIM parameters were helpful for the assessment of lymph node involvement in pancreatic carcinoma and rectal carcinoma, among which D value was the more valuable indicator for N stage [37–39]. However, previous researches have revealed that the IVIM parameters and ADC values were not related to lymph node involvement of EC [17, 25, 26]. Unlike our study, one of studies applied a simpler IVIM-MRI method with three b values (0, 400, and 1000 s/mm ^2^) to ESCC [26], so this discrepancy may have been caused by the mixing of data from different MR scanners and the number and magnitude of b values.

In this study, the D^*^ value of group T1 + T2 was significantly higher than that of group T3 + T4a (*P* < 0.05), however, no differences in D^*^ value could be found between group N + and group N0 of ESCC (*P* > 0.05). Previous study has also confirmed that the poor correlation of D^*^ value with the tumor stage [25], which might be due to its data instability, intrinsic susceptibility to noise and poor measurement reproducibility [40]. In addition to the stability and reproducibility of IVIM data, there are some potential barriers of implementing IVIM in clinical practice. For example, the uniform scanning specification for the IVIM sequence, including the number and magnitude of b-values, has not yet been reached [41, 42], which needs to be further improved by a large number of clinical practice and related research. With the continuous improvement of software availability and stability, data analysis will be more convenient, which provides the possibility for the wide application of IVIM.

Our research still had some limitations. First, the sample size in our research was relatively small. There was only one patient with T1 and T4 stages respectively, because the T1 stage of ESCC was relatively rare in clinical practice, and most cases of T1a stage undergo endoscopic mucosal resection, and T4 stage lesions are usually suggested to receive neoadjuvant chemo-/radiotherapy rather than surgery. Further study with a larger sample size may demonstrate more statistically significant results. In addition, some locally advanced ESCC received surgical resection, because some patients refused to accept neoadjuvant chemoradiation, and some cases were due to preoperative understaging. Second, due to esophageal adenocarcinoma is very rare in our country, this research included patients with ESCC only. Third, because the unresectable ESCC cannot be staged pathologically, we only evaluated the resectable lesions by using IVIM sequence. Further relevant study involving unresectable ESCC will be required to reconfirm the findings in this research. Forth, in this pilot study, only the IVIM sequence was used to evaluate the T staging and lymph node status of ESCC. The combination of conventional MR and IVIM were not performed, which might provide more effective, additional information for the T staging and lymph node metastases. In addition, they did not compare the diagnostic performance of IVIM to that of contrast-enhanced computed tomography (CECT) in the preoperative staging of ESCC. Hence, it is necessary to study the combination of multiple sequences and multiple parameters for preoperative staging of ESCC in the future.

## Conclusion

This pilot research demonstrated that IVIM sequence may be a feasible and valuable functional imaging technique to assist in the staging of primary ESCC and the prediction of lymph node metastases, which enriches the current preoperative staging methods of ESCC.

## Data Availability

Please contact authors for data requests.

## Abbreviations

ADC: Apparent diffusion coefficient
AJCC: American Joint Committee on Cancer
AUC: Area under the curve
CECT: Contrast-enhanced computed tomography
D: True diffusion coefficient
D^*^: Pseudodiffusion coefficient
DWI: Diffusion-weighted imaging
EC: Esophageal cancer
ESCC: Esophageal squamous cell carcinoma
EUS: Endoscopic ultrasonography
f: Pseudodiffusion fraction
ICC: Intraclass correlation coefficient
IVIM: Intravoxel incoherent motion
iShim: Integrated slice-specific dynamic shimming
MDCT: Multidetector computed tomography
MRI: Magnetic resonance imaging
ROC: Receiver operating characteristic
ROI: Region of interest
SNR: Signal-to-noise ratio
TSE: Turbo spin-echo
VIBE: Volumetric interpolated breath hold examination
